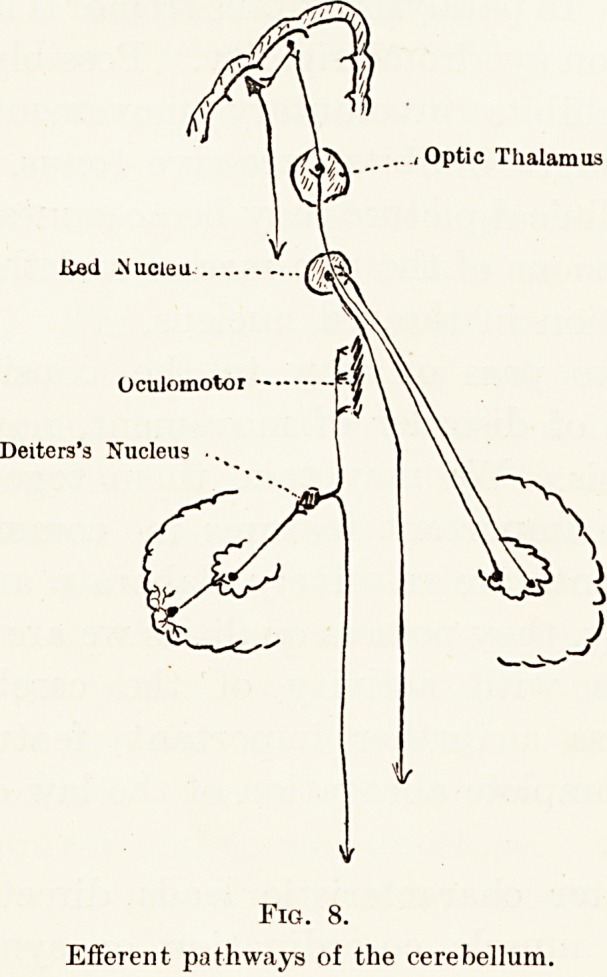# Some Disorders of Movement and Their Mechanism
*Contributed to the Bristol Medico-Chirurgical Society on 11th March, 1931.


**Published:** 1931

**Authors:** H. H. Carleton

**Affiliations:** Assistant Physician, General Hospital, and Physician, Eye Hospital, Bristol


### SOME DISORDERS OF MOVEMENT AND THEIR MECHANISM.*

BY

H. H. Carleton, M.D., M.R.C.P.,

*Assistant Physician, General Hospital, and Physician, Eye Hospital, Bristol.*

---

In practice we are frequently called upon to consider various types of motor disorder, which for purposes of diagnosis we must attempt to correlate with lesions in the nervous system. It is of some interest to know to what extent normal posture and muscle tone are determined by involuntary mechanisms within the nervous system, to distinguish disorders of movement which depend on lesions involving involuntary pathways from those which are due to disorders of pathways normally under volitional control. A complete survey of the subject is impossible within the limits of a short paper; nevertheless, some observations limited to a few clinical conditions may be of interest. In recent years a good deal of attention has been paid to certain disorders of motility, from the standpoint of altered muscle tone and posture, and the appearance of involuntary movements. Perhaps the most notable contributions have come from Kinnier Wilson in his Croonian Lectures. For our knowledge of postural reflexes we are mainly indebted to Magnus and Rademaker. The writer has drawn freely on these two sources in the present paper.

* Contributed to the Bristol Medico-Chirurgical Society on 11th March, 1931.

In the first place it is proposed to discuss muscle tone and posture, also tremor associated with striatal disease ; then briefly to consider chorea and athetosis from the point of view of synergic control of muscle action in relation to the functions of the cerebellum.

*Muscle Tone and Posture.*—In order that we may follow the problems that arise it is necessary to take a brief survey of anatomical pathways involved and consider quite shortly the physiology of muscle control, as manifest in maintenance of muscle tonus and co-ordination.

The normal individual possesses his muscles in a state of ideal tone. This depends on influences reaching the anterior horn cells of the spinal cord from different levels of the nervous system. (Fig. 1.)

FIG. 1.

Diagram modified after Tilney and Riley. Indicating various neurologica levels influencing each anterior horn cell.

The group of clinical conditions which for the sake of brevity we refer to as instances of striatal disease is characterized by alterations of posture and muscle tonus and by abnormal movements.

Let us consider the problems of altered postural tone first. To understand them it is necessary to carry the inquiry beyond the limits of the corpus striatum. For well-ordered movement to take place it is necessary to have a background of correct postural tone. The mechanism for this is subcortical, and depends on pathways closely related to the mid-brain, to the red nucleus in particular. From this level an important pathway, the rubro-spinal tract, descends to anterior horn cells of the spinal cord. In animals subjected to spinal transection at the upper level of the medulla (Fig. 2), after the

FIG. 2.
Bulbo-spinal preparation. Exhibits spinal reflexes only.

FIG. 3.
Decerebrate preparation. Exhibits static postural reflexes and extensor rigidity.

FIG. 4.
Red nucleus preparation. Exhibits righting reflexes. Extensor rigidity is absent.

period of shock is over, general rigidity ensues and certain segmental reflexes can be elicited. If the transection is made at a higher level, namely above the entrance of the vestibular nerves, but lower than the red nucleus (Fig. 3), the rigidity takes on more complex characters. It is spoken of technically as decerebrate rigidity. The animal is now capable of standing after a fashion; all the extensor muscles

(antigravitational muscles) pass into a state of firm rigidity, so that the body weight, including the head and neck, can be supported. Further, such an animal now exhibits static postural reflexes.

In animals transected as described, and in whom labyrinthine reflexes are eliminated by destruction of the labyrinth on both sides, manipulations of the neck are followed by inevitable reflex changes in the posture of the limbs. Similarly in animals with the neck muscles firmly fixed by plaster of Paris but with labyrinths intact, alteration of the spatial relations of the head in different planes is followed by postural changes in the limbs. The above changes are known as static postural reflexes; the effective stimuli which produce them arise, in the first instance, in proprioceptive impulses from the neck muscles, and have their centres in the medulla and upper cervical segments of the cord. The second class of stimuli arise in the labyrinths, and have their centres in the bulb behind the entrance of the eighth nerve.

Supposing the transection of the brain stem is carried out farther forward still, headways of the red nucleus (Fig. 4), decerebrate rigidity is absent and the muscles possess normal tone. The flexors and extensors now share equally in the state of muscle tonus. Further, the animal now exhibits what are called righting reflexes; that is to say, it is capable of restoring itself to the correct position in space if placed in any abnormal posture. An analysis of these righting reflexes is complex and a detailed consideration is outside the scope of this paper.

It should be recognized, then, that the regulation of correct muscle tonus is inherent in the mechanism of the red nucleus and the spinal tracts descending from it. An experiment of Rademaker is interesting in this connection. The rubro-spinal tracts decussate just caudal to the red nucleus in what is known as the decussation of Forel. Rademaker found that in an animal which had been transected in front of the red nucleus, and therefore possessing normal distribution of muscle tonus, section of the decussation of Forel in the median plane, which obviously must divide both rubro-spinal tracts (Figs. 5 and 6), was

FIG. 5.
Diagram of transverse section of mid-brain showing decussation of Forel below the red nucleus.

FIG. 6.
Diagram of decussation of Forel and Rademaker's median section, which produces extensor rigidity in red nucleus preparation.

immediately followed by the development of decerebrate rigidity. This experiment is fundamental, and shows the important part played by the red nucleus in the regulation of muscle tonus in animals.

It is dangerous to argue from experiments on animals as to the function of homologous neurological levels in man. Whereas in animals the rubro-spinal system is large and important, in man it is relatively insignificant. However, the experimental work, as far as it goes, establishes the principle that the regulation of muscle tonus is dependent upon an involuntary system in the region of the mid-brain. We are, however, immediately placed in difficulty when we come to consider striatal disease, in the light of physiological knowledge concerning the red nucleus. Effector pathways from the corpus striatum are relaid at the level of the red nucleus. If, therefore, the corpus striatum is destroyed by disease the rubro-spinal system is released from control. One would expect from what has gone before that no abnormal rigidity would develop, since from experiments on animals we have seen that the rubro-spinal system inhibits excessive tonus. In man, however, excessive tonus is one of the leading characteristics of striatal disease. One is forced to the conclusion that in the process of evolution in man the inhibitory function of the red nucleus as regards postural tone has been shunted forward to the striate level, in other words, that the functions of the red nucleus have, in a considerable measure at least, been superseded.

Perhaps it is desirable at this point to say a word on the subject of neurological release. We must remember that destructive lesions cannot *per se* produce positive symptoms. As Kinnier Wilson puts it, a hole in the corpus striatum cannot produce anything. If, therefore, positive symptoms appear when a certain region of the brain is destroyed, the symptoms must depend on surviving systems escaping from inhibitory control. The hypertonus of the striate patient is, therefore, an instance of a release phenomenon.

The survival of the red nucleus in striatal disease in man is therefore insufficient to secure optimum tone, and rigidity is the rule. Nevertheless, the rigidity is evenly distributed to all muscles, flexors and extensors alike, manifest clinically as so-called lead pipe rigidity. This uniformity of hypertonus is possibly due to survival of the isolated rubro-spinal system. This harmonizes with Rademaker's observations on the red nucleus preparation. This survival saves striate man from descending to the level of the decerebrate preparation in a state of extensor spasm.

Attitudinal reflexes are well seen in man in a number of pathological conditions, where the higher levels of the nervous system are cut off, as, for instance, in chronic hemiplegia, where flexion of the arm and extensor spasm of the leg are the rule. In such cases neck reflexes can be readily demonstrated as so-called associated movements. The subjects of the Parkinson syndrome tend to adopt a posture of general partial flexion. I suggest that the explanation is to be found in the light of postural reflexes seen in animals transected in front of the red nucleus. In these preparations neck flexion induces flexion of the limbs. In Parkinson's disease the patient's voluntary power is readily exhausted, he finds it difficult to hold the head up; consequently the neck droops forward under the weight of the head in spite of hypertonus of the neck muscles, subcortical postural reflexes are set up, and flexion is the characteristic static posture. In my experience the greater the flexion of the neck the greater the flexion of the arms. In the few Parkinson cases in which the head is held erect the arms tend to remain in extension by the side.

It should be noted that the cerebellum plays no part in the maintenance of postural reflexes. The experimental results in animals transected behind or in front of the red nucleus hold equally true for decerebellate animals. It would appear, then, that the rôle of the cerebellum is not manifest in the maintenance of static reflexes, in spite of its important connections with the red nucleus. It is in relation to the execution of voluntary movements that cerebellar function becomes manifest. Cerebellar lesions show themselves, on attempt at movement, as inco-ordination, intention tremor, nystagmus, etc. While the cerebellum is to be regarded as the great co-ordinating or synergic mechanism for voluntary movement, it seems probable that it also plays a part in the maintenance of optimum muscle tone, for some degree of hypotonia is characteristic of cerebellar disorders. A number of the physical signs elicited in the examination of cases with cerebellar lesions depend on this; for instance, the pendular knee-jerk, static tremor of the head, intention tremor, pass pointing, etc., etc.

As regards intention tremor, while it affords an instance of tremor occurring in hypotonic muscles, its occurrence is not really dependent upon the existence of hypotonia, but is due rather to loss of automatic muscle control. If, for instance, a patient with his arm unsupported tries to keep his finger within an inch of his nose the oscillations of intention tremor rapidly develop. Owing to faulty fixation at shoulder and elbow steady pointing is difficult. The arm sags away from its primary position, to which it is restored again by voluntary effort. The process repeats itself over and over again, so that a rhythmical tremor is established.

*Tremor.*—Tremor is a simple movement of low physiological grade in which agonist and antagonistic groups of muscle contract and relax alternately. Although seen more usually in states of muscle hypertonus, it is also seen in hypotonic muscles, and must be regarded as a phenomenon which is largely independent of muscle tone. Further, it is set at a relatively uniform rate, and appears, therefore, to be a function of neurocellular activity. It is important to note that the law of reciprocal innervation of muscles is strictly observed in tremor, in contradistinction to the disorderly inco-ordination seen in chorea and athetosis. It seems not unlikely that the ultimate mechanism of tremor belongs to the spinal level (*cf.* clonus), and that tremor occurs when this level is released from the control of a centre or centres in the mid-brain, probably in the region of the red nucleus. The release of the mid-brain mechanism is seen *par excellence* in paralysis agitans. Tremor is prone to develop in striatal lesions and lesions of the superior cerebellar peduncle. Experimentally it has been produced by stimulation of the mid-brain. On the whole, evidence tends to place the tremor controlling centre in or near the red nucleus. What particular conditions of the centre are necessary for its production it is difficult to say, but one may state that the condition of muscle tone is not an essential factor, for tremor occurs in hypertonic muscles (paralysis agitans) and in hypotonic muscles (cerebellar lesions). On the other hand, complete atonia is incompatible with the development of tremor. No totally paralysed limb trembles, and if in a limb tremor exists on account of, say, striatal disease, subsequent hemiplegia stops the tremor during the succeeding period of shock. The tremor, however, returns later when the period of shock is over and muscle tonus returns. Another point to be noted about tremor is the fact that it is never produced by a purely cortico-spinal lesion ; that is to say, the controlling mechanism is involuntary (supra-spinal) but not cortical. It requires, therefore, a destructive lesion of an involuntary system for tremor to appear as a release phenomenon. The view that the ultimate level of integration for tremor is spinal accounts for the low grade and apparently purposeless character of the movements. The control by voluntary pathways over this mechanism, released from involuntary control, is fleeting and uncertain.

A consideration of paralysis agitans on the clinical side and of Magnus's thalamus preparations (animals transected headways of the thalamus and corpus striatum) (Fig. 7) throws light on the nature of

FIG. 7.
Magnus's thalamus preparation.
No extensor rigidity, no tremor.

the tremor - controlling mechanism. The Magnus preparations with corpus striatum intact never exhibited tremor. In paralysis agitans, with the corpus striatum destroyed, tremor is a constant phenomenon. The corpus striatum consists of two separate parts, the globus pallidus and the putamen. The former is developmentally the older, and contains large motor cells. It is probably concerned with primitive automatic movements, exercising its function through the medium of the rubro-spinal system. The putamen, on the other hand, exercises inhibitory control over the primitive pallido-rubro-spinal system. In destructive lesions of the corpus striatum this primitive mid-brain motor mechanism escapes from inhibitory control and tremor develops. In Magnus's preparation with the striate body intact no tremor develops.

The very imperfect control of voluntary pathways over the released tremor-producing mechanism is seen in the temporary cessation of movement which occurs on voluntary effort, and also in the fluidity of tremor which shifts from one group of muscles to another. In paralysis agitans at one moment the movements are flexion and extension and at the next moment pronation and supination, and no voluntary effort seems able to prevent such changes.

Seeing that there is a real anatomical difference between the lesions of paralysis agitans and the Parkinson syndrome, since in the former it is essentially the corpus striatum that is destroyed and in the latter the substantia nigra, we may reasonably ascribe a difference of function to these masses, corresponding to the difference in the clinical picture of the two conditions. In paralysis agitans tremor is let loose ; in the Parkinson syndrome rigidity. Possibly the corpus striatum inhibits involuntary movements and the substantia nigra inhibits excessive tonus. The overlap in the clinical picture may be accounted for partly by mixed lesions of the two masses and their common focus of action in the red nucleus.

I wish to pass on now to the consideration of other types of disorder of movement, namely chorea and athetosis. We may take them together. They present two important features in common, namely the movements are relatively elaborate and complex, that is to say, they possess qualities we are accustomed to associate with activity of the cerebral cortex. They possess a further important feature, namely there is a complete abrogation of the law of reciprocal innervation.

This latter characteristic leads directly into the problem of muscle co-ordination or synergia. The duty of presiding over the synergic action of muscles rests entirely with the cerebellum. It is necessary, therefore, to consider in some slight detail the physiology and anatomical relations of the cerebellum.

All afferent impulses from the muscles of the body pass to the cerebellar cortex. Here they are relaid to the cerebellar nuclei, notably the dentate nucleus. Efferent tracts have their cell stations in the cerebellar nuclei and leave via the superior peduncles, with the exception of the pathways to Deiters's nucleus, which leave by the inferior peduncles. Pathways from the cerebellum form three main groups (Fig. 8) :—

1. Through the crossed rubrothalamic pathway to the frontal cortex of the opposite side.

Fig. 8.
Efferent pathways of the cerebellum.

2. Through Deiters's nucleus, forming two groups of vestibular connections :—

(*a*) Some turn headwards in the posterior longitudinal bundle, to co-ordinate vestibular impressions with eye movements.

(*b*) Some turn downwards, forming the cerebello-vestibulo-spinal tract.

3. Through the red nucleus joining the rubro-spinal pathway.

The first of these pathways interests us in connection with the mechanism of chorea and athetosis. The second will claim our attention in relation to the mechanism of nystagmus. The third is related to the part played by the cerebellum in influencing muscle tone, a subsidiary but clinically obvious function of the cerebellum.

The main physiological function of the cerebellum is synergic control of muscle action. In any voluntary movement the following functional grouping of muscles is essential: prime movers, antagonists, synergists and fixators. Without this mechanism co-ordinate muscle action is impossible.

The interaction of the cortex and cerebellum is interesting and important. Before any voluntary movement can take place there must be the idea of movement initiated in the cortex.

Messages pass back from cortex to pons. There they are relaid by the pontine cells through the middle peduncles to the cerebellar cortex, and thence back again through the dentate nucleus to the frontal cortex. These last impulses convey the delicately adjusted mechanism for muscle synergia, and co-ordinate muscular action results, exteriorized by the pyramidal tracts. In other words, we think in terms of muscle sense.

Now it is this final pathway from the cerebellum to cortex which would appear to be disorganized in chorea and athetosis. In no other way can we adequately account for the essential characteristics, namely, elaborate movements of cortical type hopelessly disorganized by the loss of synergic control. This view is the one advocated by Kinnier Wilson. It is perhaps only right to mention that other theories have been advanced to account for chorea. For instance, lesions of the thalamus have been impugned. It is to be remembered, however, that the cerebellar route through the red nucleus to the frontal cortex passes through the thalamus, consequently a thalamic lesion is liable to produce muscular asynergia, and choreic movements may appear. This, however, in no way invalidates the thesis that ordinary rheumatic chorea, which is a rheumatic encephalitis, is due to outfall of cerebellar influence on the cerebral cortex itself.

A word must be added with regard to reciprocal innervation of muscles. In tremor, essentially a subcortical phenomenon, reciprocal innervation is observed. In ankle clonus a purely spinal mechanism is released. It appears, therefore, that the ultimate integration of reciprocal innervation belongs to the spinal level. This may be true, but it in no way precludes a higher neurological level from also exhibiting control over reciprocal innervation. That this higher level may reside in the cerebral cortex is demonstrated in the phenomenon of tonic innervation. In cases of chronic hemiplegia the patient may so far recover as to be able to grip with the paralyzed hand, but is unable to release his grip to order. The same phenomenon is seen in athetosis. That is to say, his cortical control is defective, and when he attempts to use his extensor muscles the antagonistic flexors are not inhibited as they should be normally. The phenomenon of dysdiadokokinesis likewise lends support to the view that in cerebellar disease the cortex is deprived of the mechanism for quick synergic control, and in consequence there is an inability to repeat fine movements in rapid succession.

Time will not allow a separate discussion of athetosis, but the condition is so closely allied to chorea, and it is so obviously due to cortical lesions, that in the main what has been said of chorea holds good also for athetosis.

*Nystagmus.*—I propose to deal with the subject of nystagmus in a very general way, more particularly from the point of view of disordered cerebellar function, with which it is so often closely identified. Cerebellar nystagmus has as its foundation poor synergic control of the extrinsic ocular muscles. In conditions associated with cerebellar nystagmus the phenomenon is not seen when the eyes are at rest in the forward position, but is elicited on conjugate deviation. The muscles are in some degree hypotonic and synergic control poor. Consequently the new fixation point is not well held, and the eyes tend to swing back to the forward position, but are at once restored by voluntary effort to the position of lateral deviation, once more to swing back towards the middle line. There is difference in the rate of the two movements. The quick component is towards the lateral fixation point, and the slower swing back to the mid-line. In other types of nystagmus it is less easy to analyse the component movements because synergic action is not as a rule at fault. Nystagmus due to optical errors, such as anterior polar cataract, is present all the time, even with the eyes in the forward position, and vestibular nystagmus is .due to faulty integration of impulses between the labyrinthine connections and the ocular motor nuclei.

While the writer wishes to acknowledge his indebtedness to the works of Kinnier Wilson and Magnus, it should be understood that they are not in any way responsible for the views expressed in the present paper.

### BIBLIOGRAPHY.

Kinnier Wilson: Croonian Lectures, *Lancet*, July, 1925.

Magnus: Cameron Prize Lectures, *Lancet*, May, 1926.

## Figures and Tables

**Fig. 1. f1:**
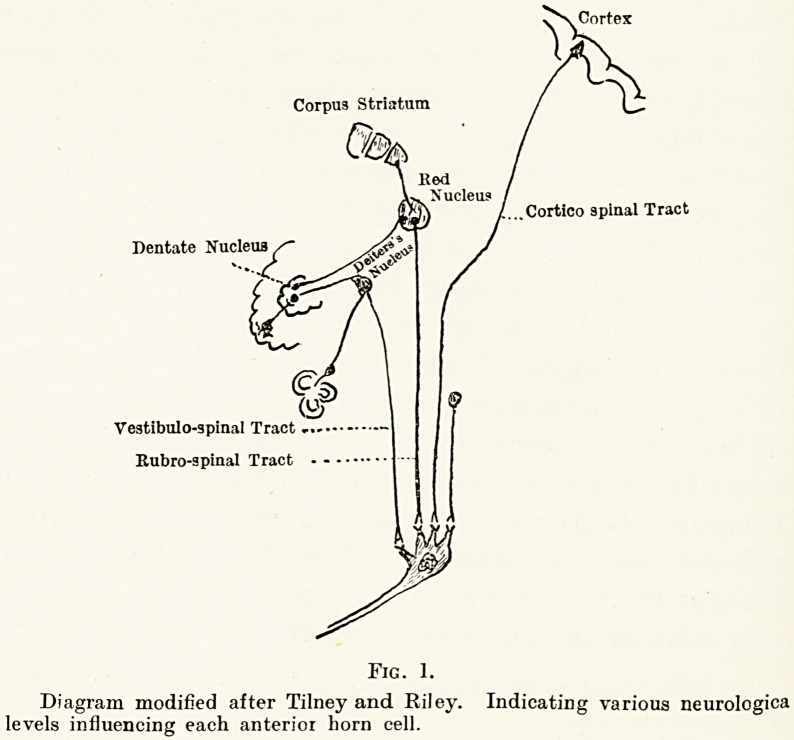


**Fig. 2. f2:**
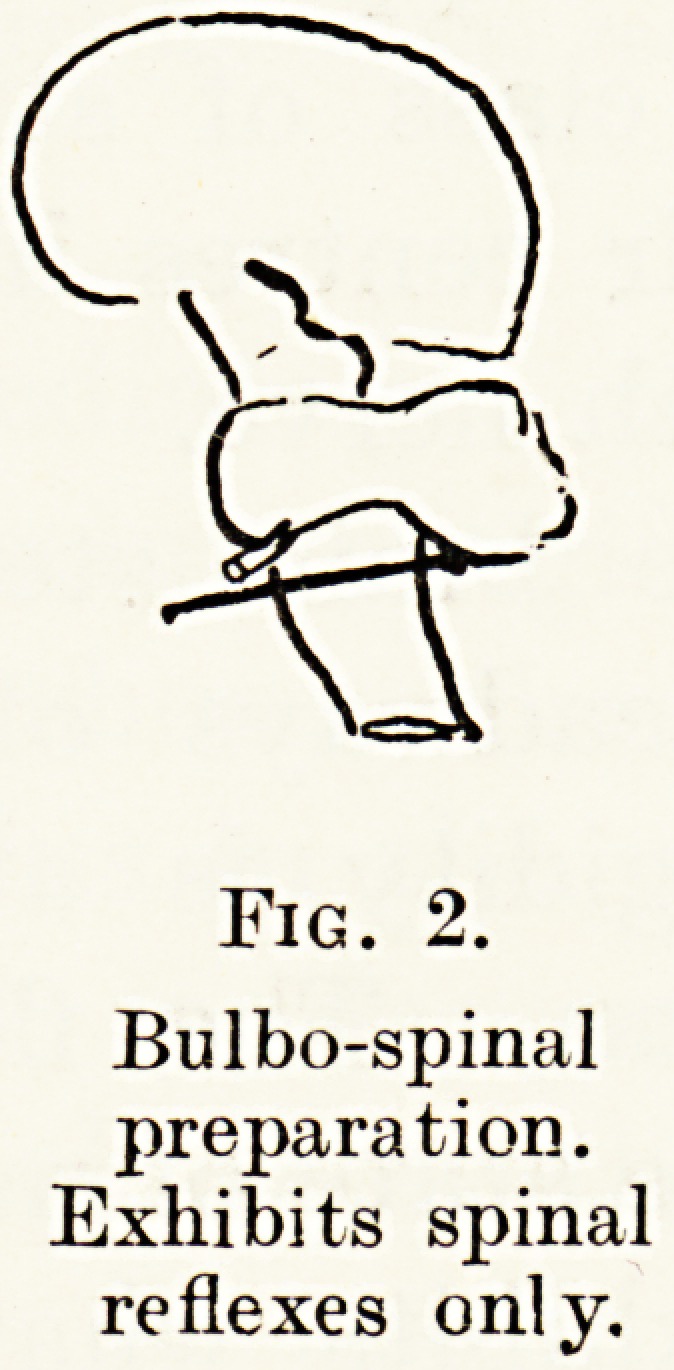


**Fig. 3. f3:**
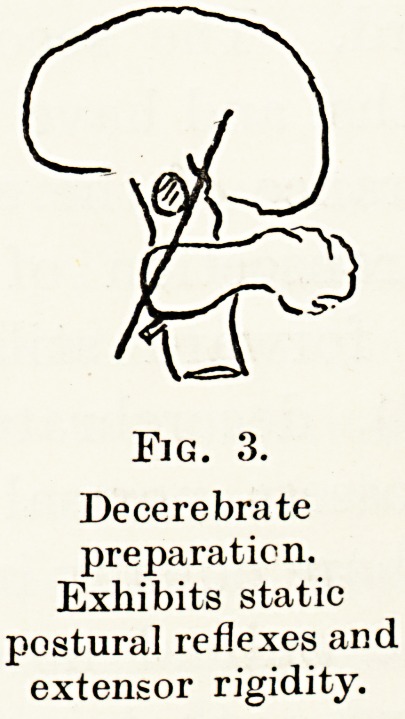


**Fig. 4. f4:**
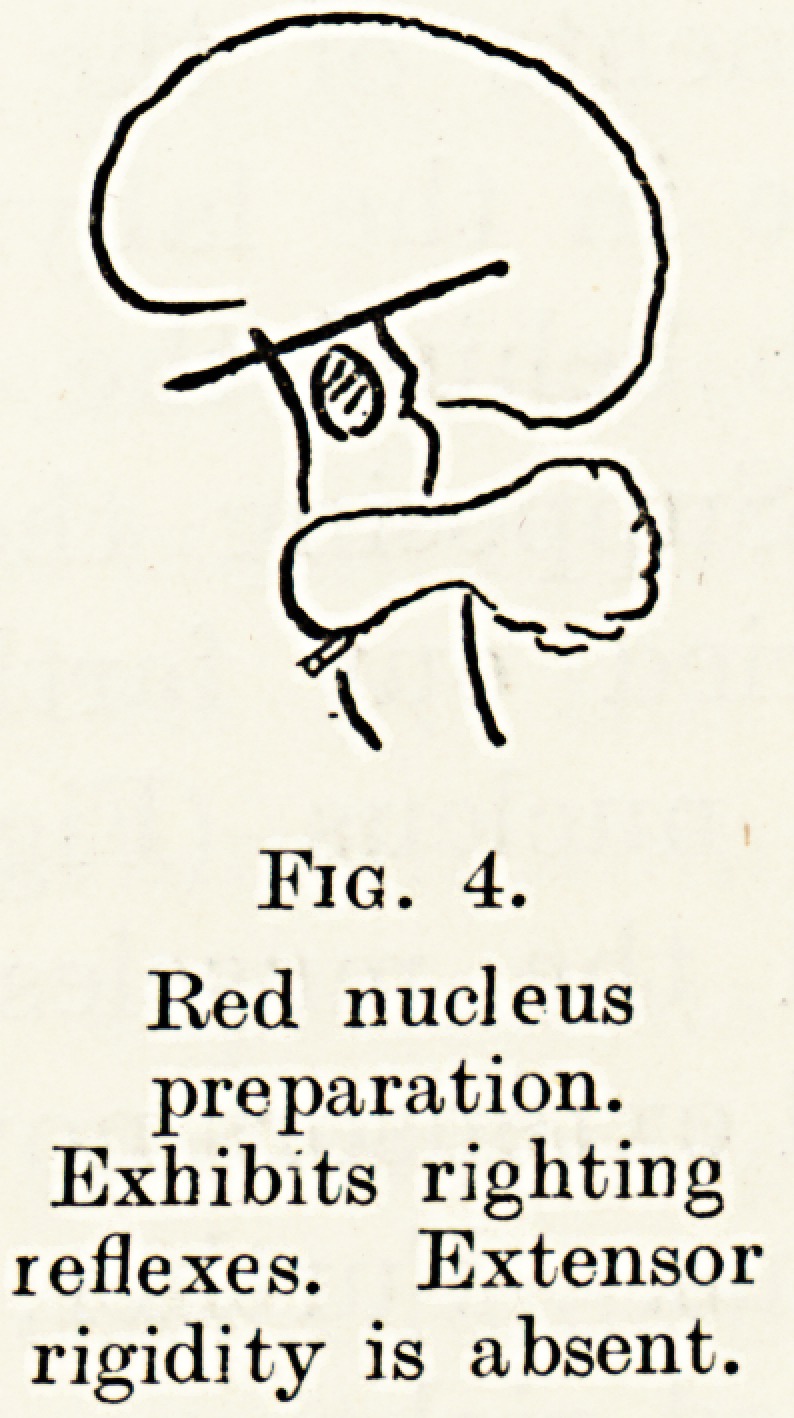


**Fig. 5. f5:**
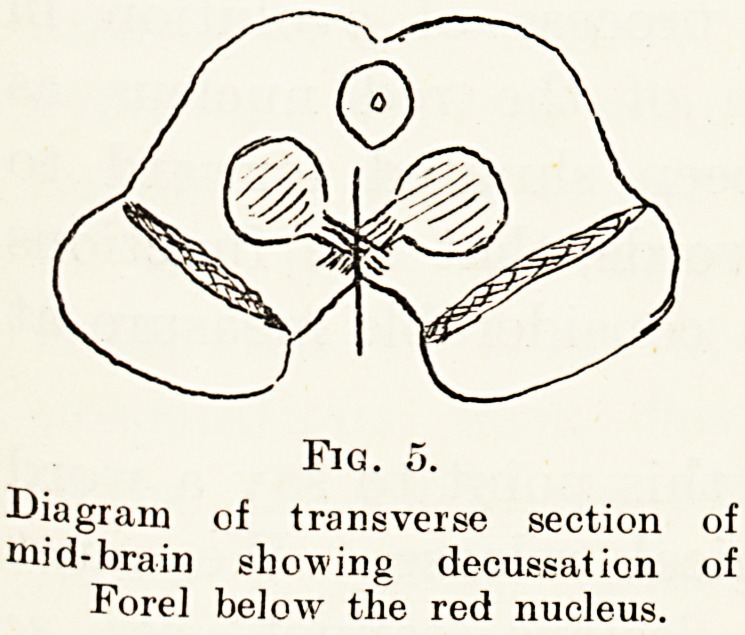


**Fig. 6. f6:**
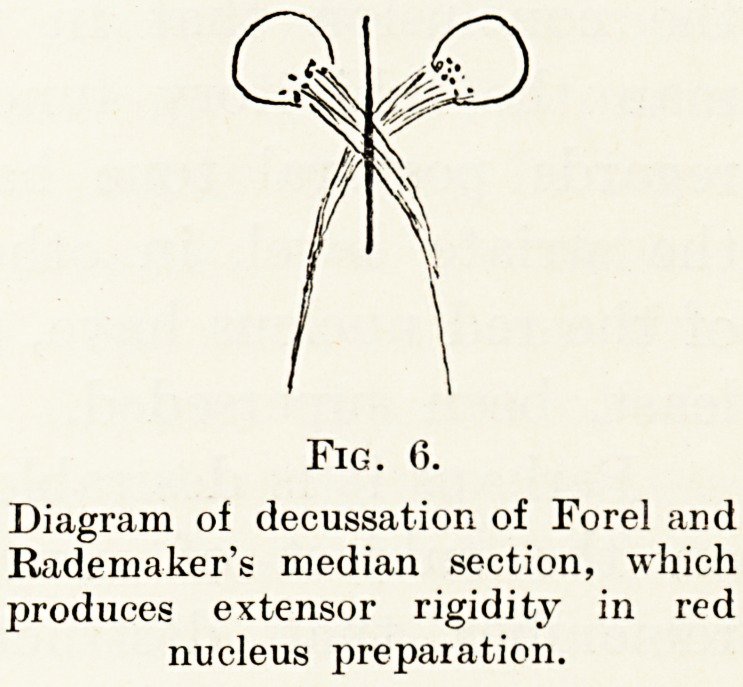


**Fig. 7. f7:**
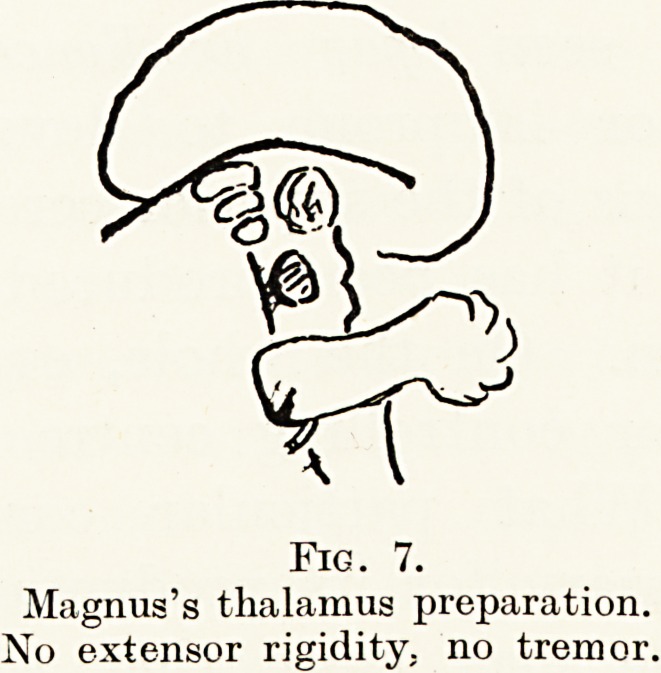


**Fig. 8. f8:**